# Development and validation of a nomogram to predict cancer-specific survival of uveal melanoma

**DOI:** 10.1186/s12886-021-01968-6

**Published:** 2021-05-25

**Authors:** Qiaozhu Zeng, Yuou Yao, Mingwei Zhao

**Affiliations:** 1 Department of Ophthalmology, Eye Diseases and Optometry Institute, Peking University People’s Hospital, No. 11 Xizhimen South Street, Beijing, Xicheng District China; 2 Beijing Key Laboratory of Diagnosis and Therapy of Retinal and Choroid Diseases, College of Optometry, Peking University Health Science Center, Beijing, China

**Keywords:** Uveal melanoma, SEER, Cancer-specific survival, Nomogram

## Abstract

**Background:**

Uveal melanoma (UM) is a rare but aggressive cancer, which is the most common primary intraocular malignancy in adults. We aimed to develop and validate a competing risk nomogram to predict cancer-specific survival (CSS) of patients with UM, as well as compare its prognostic value with that of the American Joint Committee on Cancer (AJCC) staging system.

**Methods:**

Data of patients diagnosed with UM from 2010 to 2015 were identified from the Surveillance, Epidemiology, and End Results (SEER) database. We extracted and integrated significant prognostic factors based on competing risk regression to build a nomogram. The nomogram with an online prediction version was also created. The performance of the nomogram was evaluated using Harrell’s concordance index (C-index) and calibration plots. Receiver operating characteristic (ROC) curve was carried out to estimate clinical applicability of the model. Improvements in the predictive accuracy of our new model compared with AJCC staging system were estimated by calculating the relative integrated discrimination improvement (IDI) and the net reclassification improvement (NRI).

**Results:**

A total of 839 eligible patients with primary UM were randomly assigned to a training cohort (588, 70%) and a validation cohort (251, 30%). Age, histological type, T stage and M stage were independent prognostic factors to predict CSS of UM and were incorporated in the nomogram. The calibration plots indicated that the 3- and 5-year CSS probabilities were consistent between the nomogram prediction and the actual observation. The C-index for this model was 0.778 (95% CI:0.756–0.800) and 0.786 (95% CI: 0.749–0.816) in the training cohort and validation cohort. Areas under the curve (AUCs) were 0.814, 0.771, and 0.792 in the training cohort, 0.788, 0.781 and 0.804 in the validation cohort, respectively. The NRI value in AJCC staging system was − 0.153 (95% CI -0.29 – − 0.041) for 3 years of follow-up and − 0.276 (95% CI -0.415 – − 0.132) for 5 years of follow-up. The IDI values for 3 and 5 years of follow-up in the AJCC staging system were − 0.021 (*P* = 0.076) and − 0.045 (*P* = 0.004), respectively.

**Conclusions:**

We have developed and validated a competing risk nomogram to reliably predict cancer-specific survival of patients with UM. This convenient tool may be useful for evaluating cancer-specific prognosis.

**Supplementary Information:**

The online version contains supplementary material available at 10.1186/s12886-021-01968-6.

## Background

Uveal melanoma (UM) is the most common primary intraocular malignancy in adults, with the mean age-adjusted incidence of 5.1 per million in the USA [1], representing up to 85% of ocular melanomas [2]. UM is a relatively rare but aggressive cancer, accounting for 3–5% of all melanoma cases [2]. Five-year survival rates of UM were reported to be stable as approximately 80% in the past three decades, however, up to 50% of patients may develop metastases [3, 4], Moreover, about 50% of patients with uveal melanoma might succumb to metastasis within 10 years of diagnosis. The prognosis of uveal melanoma was reported to be predicted by several independent factors, including clinical, histopathological, cytogenetic, and transcriptomic markers.

Although there have been a few articles reporting the incidence, treatment and survival of UM, the clinicopathological characteristics and prognosis of UM still need further study because of its low prevalence rate and limited cases in previous studies. In addition, no ideal prediction model for prognosis of UM was yet identified, especially for the cancer-specific survival (CSS). A nomogram is a convenient tool to predict and quantify the probability of a patient developing a certain clinical event, which was valuable for clinical decision making and risk stratification. On the other hand, the staging system for UM was changed to American Joint Committee on Cancer (AJCC) seventh edition since 2010, incorporating significant changes into the T-staging [5, 6]. Therefore, based on a large-scale population from the Surveillance, Epidemiology, and End Results (SEER) database, we are the first to develop and validate a competing risk nomogram to predict CSS of patients with UM, as well as compare its prognostic value with that of the AJCC staging system.

## Methods

### Data source and patient selection

**Fig. 1:**
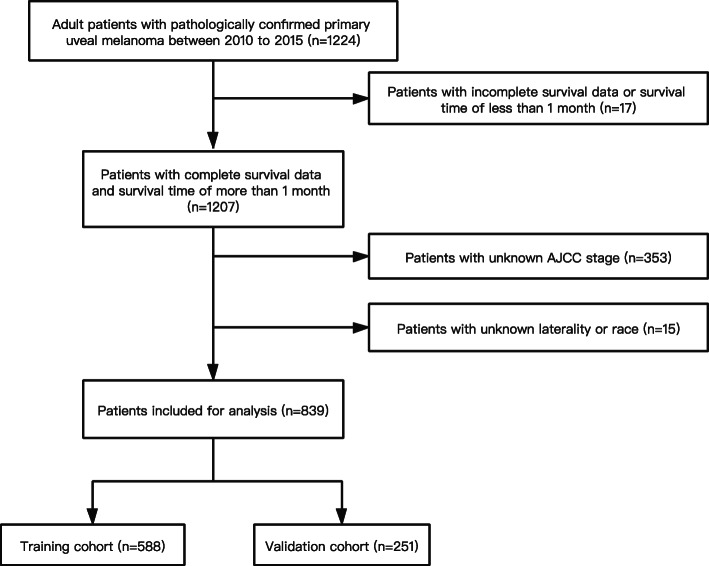
Flow chart of cases selection from SEER database

The SEER database consists of 18 population-based registries covering nearly 28% of the US population. SEER*Stat software version 8.3.6 was used to select patients from the Incidence-SEER 18 Regs Research database based on our application on November, 2020 to build the cohort. The inclusion criteria were as follows: 1) diagnosed from 2010 to 2015; 2) pathologically confirmed UM; 3) the International Classification of Diseases for Oncology-3 (ICD-3) histology code (morphology code 8720–8790); 4) ICD-O-3 site code of C69.3 (choroid) and C69.4 (ciliary body and iris); 5) only one malignant primary tumor of UM; 6) patients with active follow up and age of 18 or more than 18. We excluded patients with incomplete survival data or survival time of fewer than 1 month, unknown AJCC stage (according to the 7th edition of AJCC TNM classification), unknown laterality and race. The flow chart of patient selection was indicated in Fig. 1.

### Study variables

Several variables were extracted: baseline demographics (year of diagnosis, age at diagnosis, sex, race, insurance status, marital status), tumor features (primary site, laterality, T stage, N stage, M stage, AJCC stage, histological type, metastasis at diagnosis), therapy (surgery, radiation, chemotherapy), and survival variables (months of survival, vital status, cause-specific classification of death). CSS was the study endpoint and it was defined as the time from diagnosis to death attributed to uveal melanoma.

### Statistical analysis

In pursuit of full use of our data for constructing the predictive model, as well as keeping a considerable number of patients for its validation, we randomly allocated 70 and 30% of the enrolled eligible patients to the training cohort (*n* = 588) and validation cohort (*n* = 251), respectively, which was consistent with the assignment in some previous studies [7, 8].

The optimal cutoff values for age range were determined using the X-tile software in survival analyses. Patient characteristics were analyzed by descriptive methods, with standard summary statistics including mean (S.D.), median, interquartile range (IQR), and proportions. We performed the Student’s t test for differences for continuous, normally distributed data; continuous, non-normally distributed data were analyzed by the Mann-Whitney test. Categorical variables were processed by X2 or Fisher’s exact tests.

Fine and Gray’s competing risks regression model was applied to identify predictors of CSS. We incorporated factors with *P*-value < .05 identified in univariable analyses to develop multivariable regression models. Competing-risk regression was used to estimate the subhazard ratio (SHR) and evaluate the association between variables and risk of CCS. Death due to other causes was considered a competing event [9]. Cumulative incidence functions (CIF) for the competing event were calculated based on the competing risk methodology [10].

A nomogram was constructed based on the result of the multivariable analysis. The nomogram was subjected to 500 bootstrap resamples for internal validation with the training cohort and external validation with the validation cohort. Performance of the nomogram to predict CSS was evaluated with discrimination and calibration. The Harrell’s concordance index (C-index) was calculated to measure the discrimination ability [11], which ranges from 0.5 to 1.0, with 0.5 indicating random chance and 1.0 indicating a perfect ability to discriminate the outcome precisely [12]. The calibration was represented by a calibration curve at 3 and 5 years, comparing the predicted and observed probabilities of CSS. Receiver operating characteristic (ROC) curve was carried out to estimate clinical applicability of the model. Moreover, according to the cut-off value determined by the highest one-third of the total score calculated from the nomogram of the training cohort, all eligible patients were stratified into high- and low-risk groups, and the cumulative incidence of cancer-specific mortality curves in patients with different therapies were plotted. Improvements in the predictive accuracy of our new model were estimated by calculating the relative integrated discrimination improvement (IDI) and the net reclassification improvement (NRI) [13].

We constructed and validated the nomogram by RStudio software (V. 1.2.5001). R project’s mstate package and rms package were applied to build a nomogram, and risk Regression package was used to evaluate the performance of the nomogram. The nomogram with an online prediction version was created by DynNom package. Besides, we performed all statistical analyses by Stata/SE 15.0 (V.15.0; Stata, College Station, TX, USA), except for the development and validation of nomogram. All statistical tests were two-tailed. *P* < 0.05 was considered to be statistically significant.

## Results

### Patient characteristics

A total of 839 eligible patients with primary UM were identified from the SEER database and included in the analysis. They were randomly assigned to a training cohort (588, 70%) and a validation cohort (251, 30%). The demographic, clinicopathological characteristics and treatment information of each cohort were shown in Table 1. The median age at diagnosis of all patients were 60 years. There were 465 (55.4%) males and 374 (44.6%) females, and the majority of patients were white. Choroid was the common primary site of UM (88%). All the patients had unilateral UM, with similar rates of left or right eye involvement. Median measured basal diameter of tumor was 12 mm, with the median measured thickness of 5.4 mm. Histological types mainly included spindle cell melanoma (17.2%), mixed epithelioid and spindle cell melanoma (13.8%), epithelioid cell melanoma (5.2%) and others. The rate of metastasis to liver is 2%, which was highest among the rate of metastasis to bone, lung and brain. Most of patients possessed localized (89.6%) or regional (7.6%) historic stage. More than a half of patients with UM received radiotherapy (59%). Approximately 50% of patients underwent surgery, and only a small proportion of all patients were treated with chemotherapy (3%).

**Table 1:** Demographic, clinicopathological characteristics and treatment information of all, training and validation cohort

		All patients (*n* = 839)	Training cohort (*n* = 588)	Validation cohort (*n* = 251)	*P*-value
Age, y, median (IQR)		60 (51,69)	60 (52,69)	60 (51,68)	0.492
Age, *n* (%)					0.484
	≤58	379 (45.2)	261 (44.4)	118 (47.0)	
	> 58	460 (54.8)	327 (55.6)	133 (53.0)	
Gender, *n* (%)					0.637
	Female	374 (44.6)	259 (44.0)	115 (45.8)	
	Male	465 (55.4)	329 (56.0)	136 (54.2)	
Race, n (%)					0.352
	White	812 (96.8)	568 (96.6)	244 (97.2)	
	Black	13 (1.6)	8 (1.4)	5 (2.0)	
	Other	14 (1.7)	12 (2.0)	2 (0.8)	
Marriage, *n* (%)					0.188
	Married	467 (55.7)	326 (55.4)	141 (56.2)	
	Unmarried	296 (35.3)	202 (34.4)	94 (37.5)	
	Unknown	76 (9.1)	60 (10.2)	16 (6.4)	
Insurance, *n* (%)					0.173
	Medicaid	77 (9.2)	60 (10.2)	17 (6.8)	
	Insured	721 (85.9)	497 (84.5)	224 (89.2)	
	Uninsured	30 (3.6)	21 (3.6)	9 (3.6)	
	Unknown	11 (1.3)	10 (1.7)	1 (0.4)	
Primary site, *n* (%)					0.679
	Chorioid	738 (88.0)	519 (88.3)	219 (87.3)	
	Cilliary body and iris	101 (12.0)	69 (11.7)	32 (12.8)	
Laterality, *n* (%)					0.886
	Left	418 (49.8)	292 (49.7)	126 (50.2)	
	Right	421 (50.2)	296 (50.3)	125 (49.8)	
Histological type, *n* (%)					0.563
	Spindle cell melanoma	144 (17.2)	107 (18.2)	37 (14.7)	
	Mixed epithelioid and spindle cell melanoma	116 (13.8)	78 (13.3)	38 (15.1)	
	Epithelioid cell melanoma	44 (5.2)	29 (4.9)	15 (6.0)	
	Other	535 (63.8)	374 (63.6)	161 (64.1)	
Stage, *n* (%)					0.480
	I	159 (19.0)	117 (20.0)	42 (16.7)	
	IIa	260 (31.0)	180 (30.6)	80 (31.9)	
	IIb	172 (20.5)	125 (21.3)	47 (18.7)	
	IIIa	134 (16.0)	89 (15.1)	45 (17.9)	
	IIIb	74 (8.8)	52 (8.8)	22 (8.8)	
	IIIc	16 (1.9)	12 (2.0)	4 (1.6)	
	IV	24 (2.9)	13 (2.2)	11 (4.4)	
T stage, *n* (%)					0.976
	T1	203 (24.2)	144 (24.5)	59 (23.5)	
	T2	254 (30.3)	179 (30.4)	75 (29.9)	
	T3	236 (28.1)	163 (27.7)	73 (29.1)	
	T4	146 (17.4)	102 (17.4)	44 (17.5)	
N stage, *n* (%)					0.621
	N0	834 (99.4)	585 (99.5)	249 (99.2)	
	N1	5 (0.6)	3 (0.5)	2 (0.8)	
M stage, *n* (%)					0.150
	M0	816 (97.3)	575 (97.8)	241 (96.0)	
	M1	23 (2.7)	13 (2.2)	10 (4.0)	
Metastasis at bone, *n* (%)					0.179
	Yes	5 (0.6)	2 (0.3)	3 (1.2)	
	No	831 (99.1)	583 (99.2)	248 (98.8)	
	Unknown	3 (0.4)	3 (0.5)	0 (0)	
Metastasis at liver, *n* (%)					0.293
	Yes	17 (2.0)	9 (1.5)	8 (3.2)	
	No	819 ((97.6)	577 (98.1)	2 (96.4)	
	Unknown	3 (0.4)	2 (0.3)	1 (0.4)	
Metastasis at brain, *n* (%)					
	Yes	0 (0)	0 (0)	0 (0)	0.355
	No	837 (99.8)	586 (99.7)	251 (100)	
	Unknown	2 (0.2)	2 (0.3)	0 (0)	
Metastasis at lung, *n* (%)					0.647
	Yes	3 (0.4)	2 (0.3)	1 (0.4)	
	No	834 (99.4)	584 (99.3)	250 (99.6)	
	Unknown	2 (0.2)	2 (0.3)	0 (0)	
Historic stage, *n* (%)					0.192
	Localized	752 (89.6)	526 (89.5)	226 (90.0)	
	Regional	64 (7.6)	49 (8.3)	15 (6.0)	
	Distant	23 (2.7)	13 (2.2)	10 (4.0)	
SSF2-Measured basal diameter, mm, median		12 (8.5, 15.3)	12 (8.4,15)	12 (9,16)	0.316
SSF3-Measured thickness (Depth),mm, median		5.4 (3.1,9.5)	5.4 (3.2,9.2)	5.3 (3,9.8)	0.888
Surgery, *n* (%)					0.508
	No	450 (53.6)	311 (52.9)	139 (55.4)	
	Yes	389 (46.4)	277 (47.1)	112 (44.6)	
Radiotherapy, *n* (%)					0.451
	No	344 (41)	246 (41.8)	98 (39.0)	
	Yes	495 (59)	342 (58.2)	153 (61.0)	
Chemotherapy, *n* (%)					0.500
	No	814 (97.0)	572 (97.3)	242 (96.4)	
	Yes	25 (3.0)	16 (2.7)	9 (3.6)	

The characteristics did not significantly differ between the training and validation cohorts. The follow-up time from diagnosis to CSS was 32 (IQR: 20–51) and 32 (IQR: 18–53) months in the two groups, respectively. In the training cohort, there were 104 cancer-specific deaths (17.7%); in the validation cohorts, 55 cancer-specific deaths (21.9%) were identified.

### Independent prognostic factors in the training cohort

To identify the prognostic factors associated with CSS in the training cohort, we used the univariate and multivariate analysis based on competing-risk models (Table 2). In univariate analysis, age (*P* = 0.001), primary site (*P* = 0.026), histological type (*P* < 0.001), T stage (*P* < 0.001), N stage (*P* < 0.001), M stage (*P* < 0.001), surgery (*P* < 0.001), radiotherapy (*P* < 0.001) and chemotherapy (*P* < 0.001) were found to be significant prognostic factors. Multivariate analysis revealed that age (*P* = 0.003), histological type (*P* = 0.006), T stage (*P* < 0.001) and M stage (*P* < 0.001) were independent prognostic factors to predict CSS of UM.

**Table 2:** Univariate and multivariate analysis of factors's ability to predict CSS in the training cohort

		Univarable analysis		Multivariate analysis	
		SHR (95% CI)	*P*-value	SHR (95% CI)	*P*-value
Age			0.001*		0.003*
	≤58	Ref		Ref	
	> 58	1.748 (1.256–2.433)		1.679 (1.186–2.375)	
Gender			0.377		
	Female	Ref			
	Male	1.150 (0.844–1.567)			
Race			0.675		
	White	Ref			
	Black	0.428 (0.058–3.176)			
	Other	0.791 (0.187–3.347)			
Marriage			0.563		
	Married	Ref			
	Unmarried	1.074 (0.771–1.495)			
	Unknown	1.330 (0.786–2.253)			
Insurance			0.282		
	Medicaid	Ref			
	Insured	0.726 (0.422–1.250)			
	Uninsured	1.351 (0.551–3.311)			
	Unknown	0.916 (0.267–3.146)			
Primary site			0.026*		0.247
	Chorioid	Ref		Ref	
	Cilliary body and iris	1.568 (1.055–2.331)		1.296 (0.835–2.011)	
Laterality			0.569		
	Left	Ref			
	Right	1.093 (0.804–1.489)			
Histological type			< 0.001*		
	Spindle cell melanoma	Ref		Ref	
	Mixed epithelioid and spindle cell melanoma	3.899 (2.014–7.547)		2.593 (1.311–5.130)	0.006*
	Epithelioid cell melanoma	4.276 (1.924–9.504)		3.107 (1.315–7.301)	0.010*
	Other	2.120 (1.150–3.910)		2.128 (1.093–4.142)	0.026*
T stage			< 0.001*		
	T1	Ref		Ref	
	T2	1.478 (0.749–2.917)		1.427 (0.724–2.813)	0.305
	T3	4.389 (2.394–8.045)		4.059 (2.205–7.471)	< 0.001*
	T4	8.110 (4.464–14.736)		5.135 (2.675–9.856)	< 0.001*
N stage			< 0.001*		0.666
	N0	Ref		Ref	
	N1	10.163 (3.565–28.969)		0.770 (0.235–2.520)	
M stage			< 0.001*		< 0.001*
	M0	Ref		Ref	
	M1	15.074 (8.645–26.283)		11.531 (5.115–25.992)	
Surgery			< 0.001*		0.961
	No	Ref		Ref	
	Yes	1.869 (1.366–2.556)		1.018 (0.511–2.028)	
Radiotherapy			< 0.001*		0.479
	No	Ref		Ref	
	Yes	0.512 (0.376–0.698)		0.776 (0.385–1.565)	
Chemotherapy			0.023*		0.213
	No	Ref		Ref	
	Yes	2.301 (1.119–4.731)		0.512 (0.179–1.467)	

*P < 0.05

### Prognostic nomogram for cancer-specific survival of uveal melanoma

**Fig. 2:**
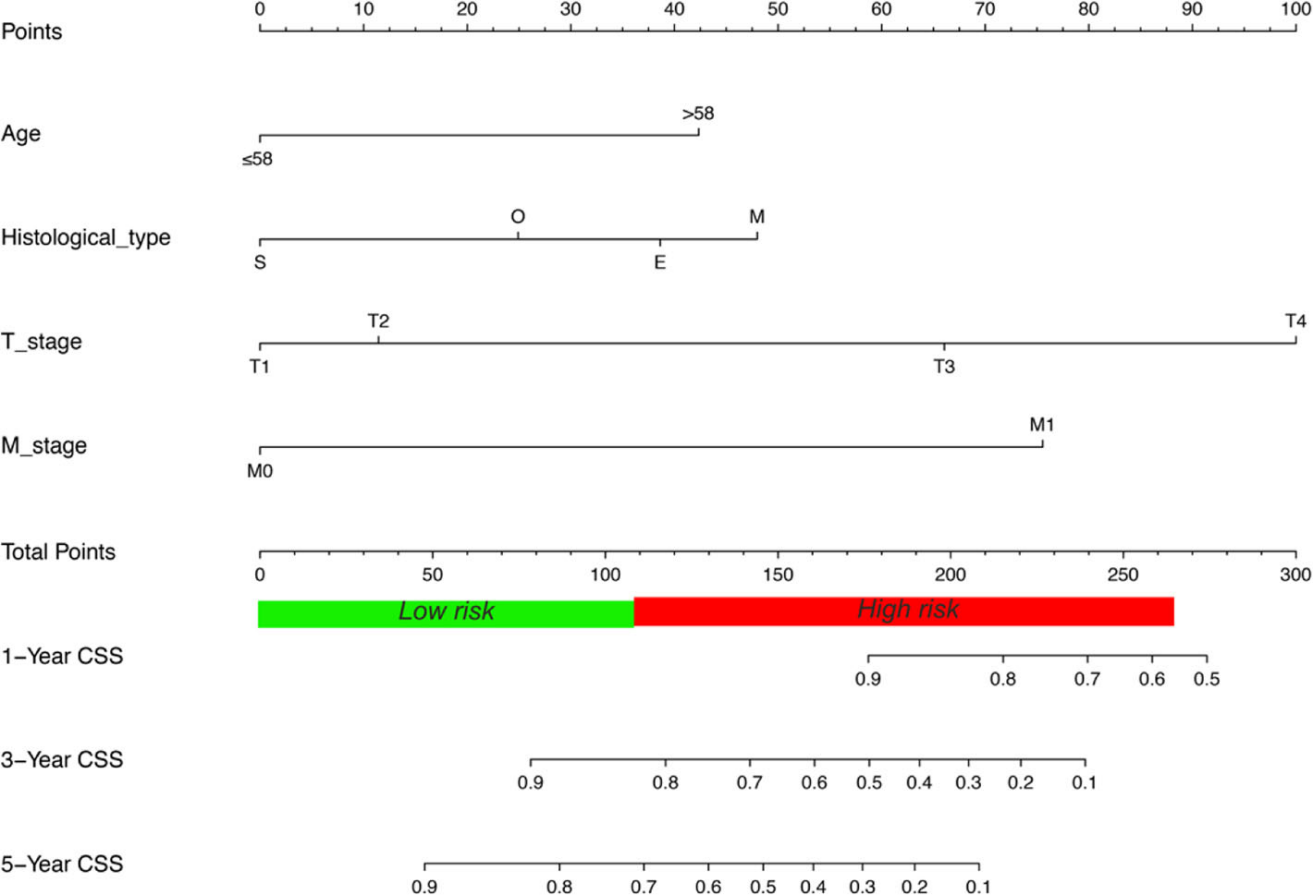
Prognostic nomogram predicting the probability of 3- and 5-year CSS in patients with uveal melanoma. The total scores of independent prognostic factors projected to the bottom scale represent the probabilities of 3- and 5-year CSS

All independent prognostic factors were included to develop a nomogram to calculate the 3-year and 5-year CSS probabilities (Fig. 2). From the nomogram, T stage was found the most predominant contributor to the prognosis. Each subtype within these four significant independent variables was assigned a score on the point scale. The total scores of independent prognostic factors projected to the bottom scale represent the probabilities of 3- and 5-year CSS.

### Calibration and validation of the nomogram

**Fig. 3:**
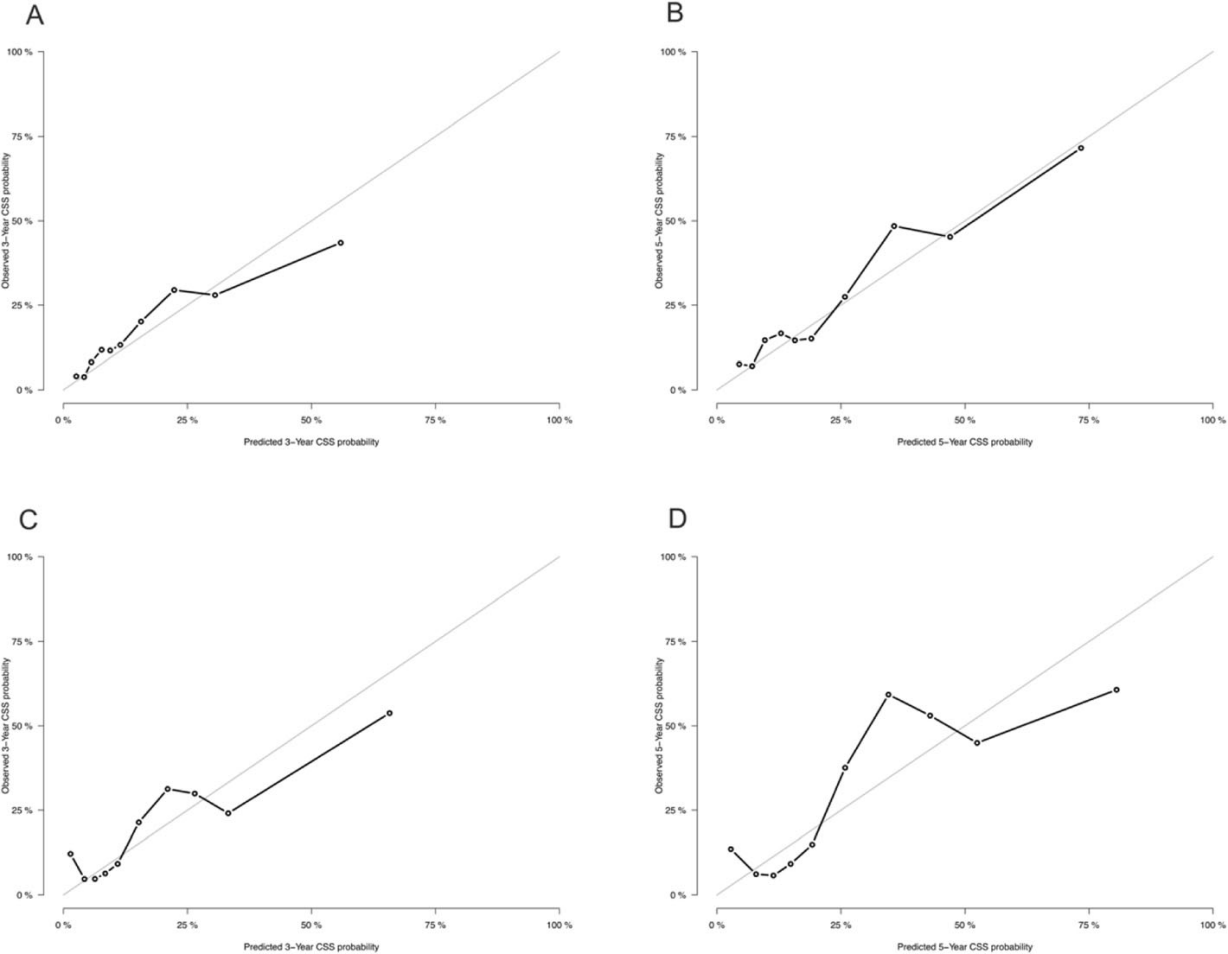
The calibration curves for predicting CSS in the training cohort **a, b** and validation cohort **c, d** at the time point of 3 and 5 years respectively. Nomogram-predicted probability is plotted on the x-axis; actual probability is plotted on the y-axis

**Fig. 4:**
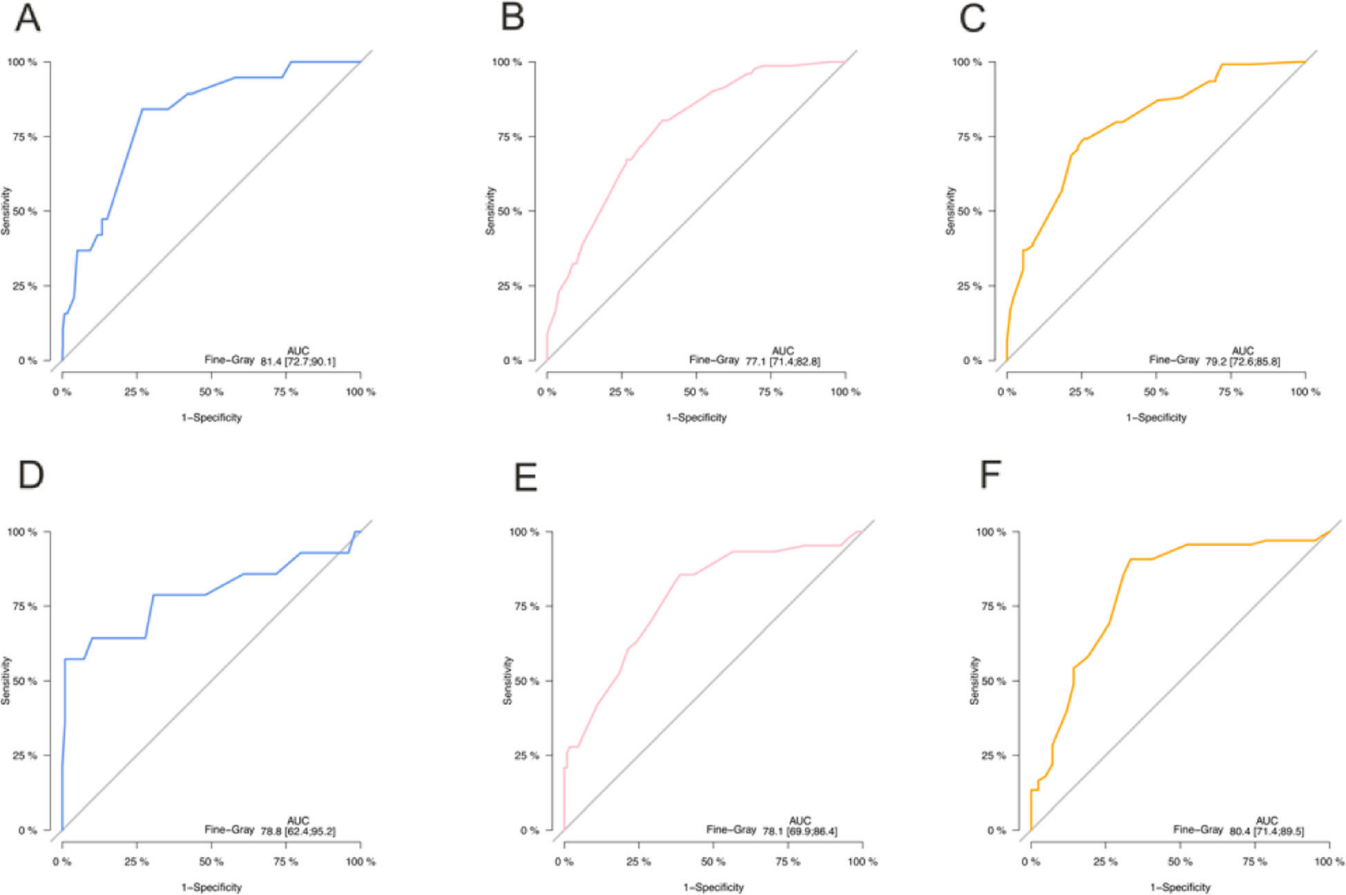
ROC curves to verify accurate predictability for 1-, 3- and 5-year CSS in the training cohort **a, b, c** and validation cohort **d, e, f**

Our nomogram was tested by 500 bootstrap resamples for the internal validation with the training cohort, as well as the external validation with the validation cohort. Calibration plot for predicting 3- and 5-year CSS of UM demonstrated a good agreement between the predicted and actual CSS probabilities in both the internal and external validations (Fig. 3). The Harrell’s C-index for this model was 0.778 (95% CI:0.756–0.800) and 0.786 (95% CI: 0.749–0.816) in the training cohort and validation cohort. We performed ROC curves to verify accurate predictability for 1-, 3- and 5-year CSS. Areas under the curve (AUCs) were 0.814, 0.771, and 0.792 in the training cohort, 0.788, 0.781 and 0.804 in the validation cohort, respectively (Fig. 4). The nomogram was programmed into an online calculator, which is available at https://zengqiaozhu123.shinyapps.io/UM_prognosis/.

### Nomogram performance in risk group stratification

**Fig. 5:**
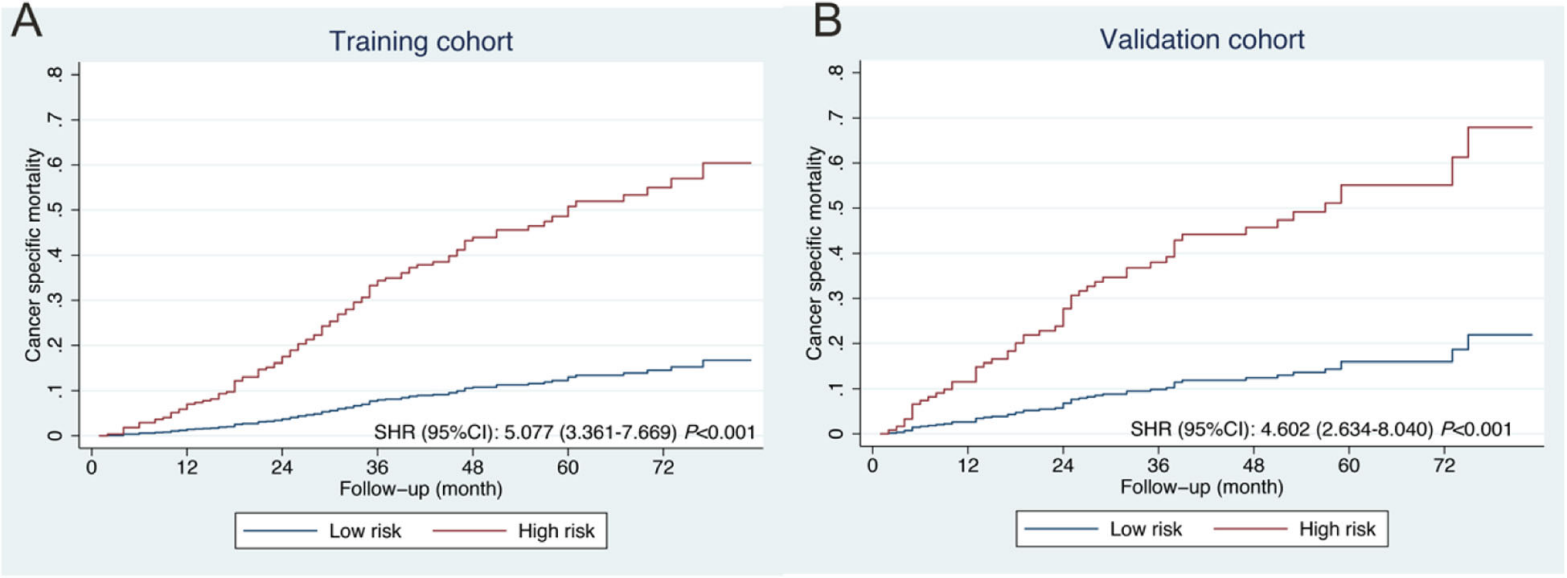
Cumulative incidence curves of UM cancer-specific mortality in the training **a** and validation **b** cohorts

Consistent with some previous studies, the cut-off value was determined from top one-third of the total scores of patients in the training cohort calculated from the nomogram. Patients were classified into low risk (*n* = 388, 66.0%, score < 108) and high risk (*n* = 200, 34.0%, score ≥ 108) groups. Distinct cumulative incidence curves of UM cancer-specific mortality were depicted in the training and validation cohorts (Fig. 5).

**Fig. 6:**
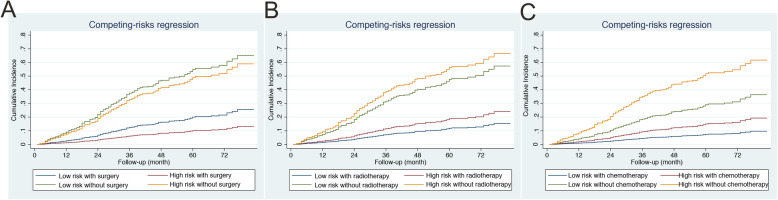
Cumulative incidence of mortality of high-risk and low-risk groups in patients treated with surgery **a**, radiation **b** or chemotherapy **c**

**Fig. 7:**
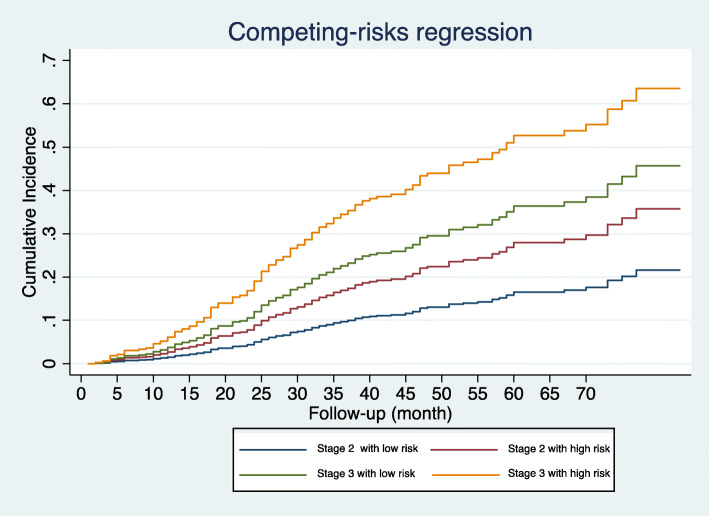
Cancer specific mortality of patients with different AJCC stage of 2 and 3 in different risk groups

Patients treated with surgery, radiation or chemotherapy were categorized into the high-risk and low-risk groups (Fig. 6). For surgery, in high-risk groups, no significant difference in cancer specific mortality was found between surgery and no surgery; while in low-risk group, surgery could statistically reduce the mortality (SHR 0.478, 95% CI 0.275–0.831, *P* = 0.009). For radiation, there was no difference in cancer specific mortality between radiation and no radiation in the high-risk or low-risk groups. In terms of chemotherapy, there was also no difference in cancer specific mortality between chemotherapy and no chemotherapy in the high-risk or low-risk groups, indicating that the nomogram appears to be able to differentiate patients with a different risk. In addition, we compared the cancer specific mortality of patients with different AJCC stage of 2 and 3 in different risk groups. In low-risk or high-risk group, there was no difference in mortality between patients with AJCC stage 2 and 3; while high-risk group presented with higher cancer specific mortality than low-risk group regardless of the AJCC stage (Fig. 7).

The NRI value in AJCC staging system was − 0.153 (95% CI -0.29 – − 0.041) for 3 years of follow-up and − 0.276 (95% CI -0.415 – − 0.132) for 5 years of follow-up. These results indicate that our new model exhibited markedly superior predictive performance compared to the AJCC model. Similarly, the IDI values for 3 and 5 years of follow-up in the AJCC staging system were − 0.021 (*P* = 0.076) and − 0.045 (*P* = 0.004), respectively.

## Discussion

UM is a rare but aggressive intraocular malignancy. Despite advances in the local treatment methods for primary UM, the survival rates have not improved [14]. To date, there was no available prognosis prediction model for UM, especially model of CSS. Herein, we developed and validated a personalized nomogram predicting the CSS of UM patients, using a large SEER cohort. Our results supported the satisfactory performance of the nomogram in UM prognosis prediction, and compared it with AJCC staging system. The patients stratified into different risk groups demonstrated distinct cancer-specific mortality curves within respective therapies.

There have been numerous factors identified to predict overall survival of UM, including clinical features (tumor size), histopathological features (epithelioid cell type, mitotic activity, HLA expression, tumor infiltration by proangiogenic M2-macrophages and lymphocytes, microvascular loops and networks, and extracellular matrix patterns), cytogenetic features (abnormalities on chromosomes 3, 6, 8 and 1) and etc. [15]. Xu et al. investigated the SEER database (2010–2015) and found that determinants of overall survival and CSS included age at diagnosis, AJCC stage, and radiation therapy [16]. Similarly, in Mahendraraj et’s study based on SEER database (1973–2012), it was proposed that male sex, older age, distant disease, and primary surgical therapy rather than radiotherapy are associated with an increased risk of mortality [17]. The compare and contrast table of different studies about the UM prognosis based on SEER database were indicated in Supplementary Table 1. In a retrospective study incorporating 171 UM patients by Yue et al., a large basal tumor diameter, ciliary body involvement, non-spindle cell type, extrascleral extension, and negative BAP1 staining were risk factors for the prediction of UM prognosis [18]. Coleman et al. also found that a combination of cell type, glaucoma, and largest tumor diameter had strong and independent prognostic significance for UM [19]. In some larger cohorts, it was proposed that older age at diagnosis and male gender were correlated with increased mortality [20–23]. However, almost all of those studies were in the method of Cox regression to investigate the OS or CSS, with relatively limited sample sizes. Consistent with previous findings, our results showed that age, T stage categories, M stage categories, and the histological types were independent prognostic factors to predict CSS of the patients with UM. In univariate analysis, radiotherapy was a protective factor for CSS. Conversely, surgery and chemotherapy were found to be risk factors for CSS. We speculated that patients receiving surgery or chemotherapy might have been burdened with more severe condition, and heterogeneity of inclusion criteria, methods/types of surgeries or chemotherapies in different institutions may explain for the result. We observed no statistical significance of surgery, radiotherapy or chemotherapy in the multivariate analysis. In addition, although the AJCC staging system is a significant predictive tool for the prognosis of UM, it lacks of some important risk factors such as age, sex, and marital status. To our knowledge, our study first built a nomogram to predict CSS of UM, which may act as a supplement to the prognosis of UM using the AJCC staging system. The constructed nomogram exhibited good discrimination and calibration, with C-index and AUC of more than 0.7.

Based on our nomogram, we developed a risk classification system that excellently classified patients with UM into high- and low-risk of cancer-specific death. In low-risk group, surgery could statistically reduce the mortality, suggesting the significant positive impact of surgery on patients with low risk. For radiation/chemotherapy, no difference in cancer specific mortality was found between radiation/chemotherapy and no radiation/chemotherapy whether in the high-risk or low-risk groups, indicating that patients were categorized into distinct risk groups regardless of the treatment. With limited information on detailed treatment from SEER database, we should cautiously interpret the influence of different therapies on prognosis. Management of UM mostly depends on the site, size of tumor and local extension [24], Treatment aims at preserving the eye and useful vision and preventing metastases. In USA, there has been a trend from local resection and enucleation into radiotherapy [14]. However, the long-term vision loss of radiation is still inevitable. For metastatic UM, liver-directed therapy and systemic therapy (chemotherapy, immunotherapy, targeted therapy) have been utilized. Besides, a lot of advanced approaches are currently under research, such as selumetinib, tebentafusp and etc. From the biological view, prevention and/or treatment should be based on the specific mutation of the tumor of the patients in the future. Further prospective large studies are required to investigate the impact of novel treatments on UM patients [25].

Regardless of the AJCC stage, high-risk group presented with higher cancer specific mortality than low-risk group, indicating that our model appears to be able to efficiently differentiate patients with a different risk. Moreover, we introduced two indexes of NRI and IDI. NRI reflects how the nomogram reclassifies the risk probabilities better than the AJCC staging system, and IDI indicates the improvement in the ability of the nomogram to distinguish between AJCC stages. Compared with AJCC staging system, our new model exhibited markedly superior predictive performance. Considering our model couldn’t incorporate all prognostic factors for clinical decision, more clinical researches were needed to validate its applicability.

Meanwhile, there are several limitations in our study. First, the retrospective nature of SEER-based study may bring about biases. Some crucial data, including lymphovascular invasion, detailed information of the tumor, detailed treatment approaches, some relevant molecular or cytogenetic factors and information of the 8th edition of AJCC staging system were not available, restricting further analysis. Cytogenetic studies are currently at the forefront of uveal melanoma management. In our clinical practice, it is not easy to obtain cytogenetic data, which requires more investment and support. We will take this indicator and more other factors into consideration in our further research, to construct a more advanced prediction model. Additionally, we could not neglect the geographical bias of the nonuniform distribution of the SEER registries, including the race heterogeneity. Second, because of the low disease prevalence, the cohort was relatively small. Larger sample sizes are necessary for further independent validation and prognostic stratification analysis. Third, we only included patients with complete information, which would have introduced selection bias. Finally, although our nomogram and risk classification system were constructed and validated in two independent subgroups from the SEER database, external validation with other populations is still warranted.

## Conclusions

In conclusion, we have developed and validated a competing risk nomogram to reliably predict cancer-specific survival of patients with UM, of which the prognostic value was better than that of the AJCC staging system alone. This convenient tool may be useful for evaluating cancer-specific prognosis, identifying patients with high-risk of cancer-specific death and clinical decision making. Future external validation and larger prospective studies are still required.

## Supplementary Information

**Additional file 1: Supplementary Table 1.** The compare and contrast table of different studies about the UM prognosis based on SEER database.

## Data Availability

The data were abstracted from an open database, the Surveillance, Epidemiology, and End Results (SEER) database (https://seer.cancer.gov). Data described in the manuscript, accession number, and password will be made available by the corresponding author upon request.

## Abbreviations

UM: Uveal melanoma
CSS: Cancer-specific survival
SEER: The Surveillance, Epidemiology, and End Results database
C-index: Harrell’s concordance index
ROC curve: Receiver operating characteristic curve
IDI: Integrated discrimination improvement
NRI: The net reclassification improvement
AJCC: The American Joint Committee on Cancer
AUCs: Areas under the curve
SHR: Subhazard ratio
CIF: Cumulative incidence functions
IQR: Interquartile range
