# Supplementary material for: Development and validation of a nomogram to predict cancer-specific survival of uveal melanoma
Source: BMC Ophthalmol. 2021 May 25;21:230. doi: 10.1186/s12886-021-01968-6 (PMC8147099; doi:10.1186/s12886-021-01968-6)
Supplement: Supplementary file 1 — Additional file 1: Supplementary Table 1. The compare and contrast table of different studies about the UM prognosis based on SEER database. [file 12886_2021_1968_MOESM1_ESM.docx]

**Supplementary Table 1 The compare and contrast table of different studies about the UM prognosis based on SEER database**

| No. | Author | Year of publish | n | Year | Outcomes | Regression model | Nomogram | Factors |
| --- | --- | --- | --- | --- | --- | --- | --- | --- |
| 1 | Mahendraraj, K., et al. | 2016 | 7516 | 1973-2012 | Mortality | NR | No | Male sex, age >50 years, distant metastases, and surgical treatment only as independently associated with increased mortality. Hispanic ethnicity and radiation treatment were associated with reduced mortality. |
| 2 | Rao, Y. J., et al | 2017 | 2611 | 2004-2013 | Disease-specific survival (DSS), overall survival (OS) | Cox | No | Enucleation was associated with inferior DSS and OS compared to globe preserving therapy. Limited surgical resection or ablation and radiation had similar DSS and OS. |
| 3 | Cai, W., et al | 2020 | 4217 | 2004-2015 | Cancer-specific survival, OS | Cox | No | Marital status was proved to be an independent prognostic value for survival in UM patients |
| 4 | Andreoli, M. T., et al. | 2015 | 7043 | 1973-2009 | DSS and OS | Cox | No | Tumor histology, T stage and age at diagnosis were associated with DSS |
| 5 | Xu, Y., et al. | 2020 | 1142 | 2010-2015 | Five-year accumulative OS, and DSS | Cox | No | AJCC stage, and radiation therapy were found to be consistent predictors in both univariate and multivariate analysis models. |

NR, not reported
